# Deactivation of CK1α enhances the anti-cancer effects of salinomycin in colorectal cancer HCT116 cells

**DOI:** 10.1016/j.bbrep.2025.102293

**Published:** 2025-10-29

**Authors:** Sara Khakshournia, Morvarid Siri, Mozhdeh Zamani, Farzaneh Bozorg-Ghalati, Zahra Mojtahedi, Somayeh Igder, Negar Azarpira, Pooneh Mokarram

**Affiliations:** a Autophagy Research Center, Shiraz University of Medical Sciences, PO Box: 71345-1849, Shiraz, Iran; b Department of Biochemistry, School of Medicine, Shiraz University of Medical Sciences, PO Box: 71345-1849, Shiraz, Iran; c School of Public Health, University of Nevada, PO Box: 89154, Las Vegas, NV, 89154, USA; d Department of Clinical Biochemistry, School of Medicine, Ahvaz Jundishapur University of Medical Sciences, PO Box: 61355- 45, Ahvaz, Iran; e Autophagy Research Center, Department of Biochemistry, School of Medicine, Shiraz University of Medical Sciences, PO Box: 71345-1849, Shiraz, Iran

**Keywords:** Salinomycin, Casein kinase 1 alpha, Autophagy, Ferroptosis, Colorectal cancer, HCT116 cells

## Abstract

Salinomycin (Sal), an ion-carrier antibiotic, effectively suppresses cancer growth and metastasis through autophagy or ferroptosis induction, thereby overcoming drug resistance. D4476, a selective inhibitor of casein kinase 1 alpha (*CK1α*), also inhibits the growth of tumors. However, their combined effect on colorectal cancer (CRC) cell growth and the mechanism underlying this effect remain unknown. This study evaluated the impact of Sal and D4476 on HCT116 CRC cells growth, ferroptosis, and autophagy by utilizing MTT assays, real-time PCR, Scratch Wound Healing Assay, reduced glutathione (*GSH*), and lipid peroxidation assays. It was discovered that Sal in combination with D4476 inhibited cell growth and triggered ferroptosis in a time- and dosage-dependent way, with nuclear factor *E2*-related factor 2 (*NRF2*) expression decreasing. Nevertheless, the level of phosphohydroxythreonine aminotransferase 1 (*PSAT1*) expression is higher in the Sal-D4476 combination compared to Sal alone. In addition, this combination resulted in a synergistic depletion of *GSH* and production of *MDA*, as well as an inhibition of autophagic flux by upregulating the gene expressions of *Beclin1*, *LC3βII*, and *p62*. In conclusion, the combination of Sal and D4476 suppressed the growth of HCT116 CRC cells by inducing ferroptosis and inhibiting autophagic flux. This research could lead to a novel method of using Sal in the clinic as a new antitumor drug, particularly when combined with other therapies that target the *p62-NRF2* axis, such as D4476.

## Highlights

• The combination of salinomycin and D4476 markedly inhibited HCT116 cell proliferation, exceeding the effects of each compound individually.
• Salinomycin and D4476 induced ferroptosis, diminishing the expression of NRF2 and PSAT1.
• The integrated therapy markedly diminished autophagic flux.
• Salinomycin and D4476 may obstruct the antioxidant p62-NRF2-PSAT1 pathway.

## Abbreviations:

CRC: Colorectal cancer
MDA: Malondialdehyde
GSH/GSSG: reduced glutathione to oxidized glutathione
LC3: microtubule-associated protein 1 light chain 3
Keap1: Kelch-like ECH-associated protein 1
NRF2: nuclear factor E2-related factor 2
SHMT2: Serine Hydroxymethyltransferase 2
HGDH: hydroxyglutarate dehydrogenase
PSAT1: phosphohydroxythreonine aminotransferase 1
Sal: Salinomycin
BCSCs: breast cancer stem cells
mTOR: mammalian target of rapamycin
CK1α: casein kinase 1 alpha
UPR: unfolded protein response
FBS: fetal bovine serum
2-D: two-dimensional
TNB: 2-nitro-5-thiobenzoate
NPMI: N-methyl-2-phenylindole
SD: standard deviations
EMT: epithelial-mesenchymal transition
ECM: extracellular matrix
TCF4: T cell-specific transcription factor
GPX4: Glutathione peroxidase 4
4-HNE: 4-Hydroxynonenal

## 1 Introduction

Colorectal cancer (CRC) is the most prevalent cancer of the digestive tract, accounting for over 90 % of all colon tumors. To date, CRC is the third leading cause of cancer-related deaths in the world, with a poor 5-year survival rate. The incidence of CRC is anticipated to rise in the future as a result of changes in lifestyle, such as dietary patterns, alcohol abuse, and smoking [[1], [2], [3]]. The effectiveness and outcomes of CRC treatment depend heavily on the disease's stage at the time of diagnosis. Currently, the only viable treatment for metastatic CRC is adjuvant chemotherapy following surgical resection, but only 10 %–15 % of patients have curable disease [4,5]. For incurable CRC, chemotherapy-induced cancer cell death is a crucial therapeutic option.

Cell death is a genetically regulated process of cell suicide that has apoptotic and nonapoptotic forms with distinct mechanisms [6]. Compared to conventional anticancer drugs that induce apoptosis, agents that induce non-apoptotic cell death have recently introduced new treatment alternatives for CRC. In particular, their ability to induce ferroptosis-mediated cellular death in CRC has sparked considerable interest [7,8]. Ferroptosis is an innovative type of non-apoptotic cell death marked by a rise in iron (II) levels, lipid peroxidation products like malondialdehyde (MDA), and a decline in the ratio of reduced glutathione to oxidized glutathione (GSH/GSSG) [9]. Studies have shown that ferroptosis is essential for the development of CRC, and an abundance of ferroptosis can suppress CRC proliferation and invasion via an imbalance of iron homeostasis, accumulation of lipid peroxides, and disruption of the glutathione/glutathione peroxidase equilibrium [10].

With the in-depth investigation of ferroptosis, pertinent research has revealed that ferroptosis is not an independent phenomenon and has close ties to other cellular processes [11,12]. Surprisingly, new insights indicate that the induction of ferroptosis is dependent on autophagy, which is a lysosomal degradation pathway accountable for recycling damaged cell constituents during survival stress [13,14]. Autophagy can contribute to ferroptosis by eliminating lipid oxidation via the activation of the protein Sequestosome 1 (*p62/SQSTM1)*- NRF2 (nuclear factor *E2*-related factor 2) axis [15]. The p62-NRF2 axis is a redox- and xenobiotic-sensitive pathway whose function is to protect normal or cancerous cells from oxidative stress and controlled by posttranslational modifications including sequential phosphorylation and ubiquitination of p62 [16]. The p62 is a prototypical autophagy receptor that binds ubiquitinated cargos to the autophagy machinery by direct interaction with microtubule-associated protein 1 light chain 3 (LC3), allowing selective substrate deterioration [17]. Under stress condition(s), phosphorylation of p62 activates NRF2 signaling by sequestering Keap1 (Kelch-like ECH-associated protein 1), leading to the upregulation of cytoprotective antioxidant genes including Serine Hydroxymethyltransferase 2 (SHMT2), hydroxyglutarate dehydrogenase(HGDH), and phosphohydroxythreonine aminotransferase 1 (PSAT1), thereby protecting cancer cells from oxidative stress [18]. Recent researches indicate that p62-NRF2 signaling activation induces tumorigenesis in precancerous cells and promotes the growth and therapy resistance of tumor cells in human hepatocellular carcinomas (HCC) [19,20], lung adenocarcinoma [20,21], and CRC [22]. Moreover, they demonstrated that inhibition of p62-NRF2 signaling can promote the ferroptosis process in CRC, indicating that p62 and NRF2 have anti-ferroptosis functions [23]. Therefore, inducing ferroptosis through interrupting NRF2/p62 interplay can emerge as a therapeutic strategy for inducing cancer cell death in CRC tumors that are drug-resistant.

Salinomycin (Sal) is an antibiotic that was introduced as a novel and effective anti-cancer drug with pro-ferroptosis effects [24]. Research demonstrates that Sal and its derivative, ironomycin, effectively eliminate breast cancer stem cells (BCSCs) via suppression of p62-NRF2 antioxidant signaling. This is accomplished by sequestering iron in lysosomes, which induces the overproduction of ROS and permeabilization of the lysosomal membrane, therefore promoting ferroptosis [25,26]. It has been also suggested that Sal could serve as a promising treatment by inhibiting the *p62-NRF2* interaction, and *NRF2* activity and promoting ROS production in nasopharyngeal and prostate cancer cells [27,28]. On the other hand, Sal has been found to play a significant role in inducing CRC cell death and autophagy flux inhibition by primarily targeting the unfolded protein response (UPR) and autophagy pathways [29]. Also, prior studies provide evidence that Sal stimulates cell death with autophagy flux inhibition through activation of endoplasmic reticulum stress in human NSCLC [30] and glioblastoma cancer cells [31], which may represent a potential therapeutic approach for cancer treatment. Moreover, studies indicate that Sal is instrumental in inhibiting p62-NRF2 antioxidant signaling, leading to the enhancement of ferroptosis and autophagy flux inhibition [28]. Then, combining Sal with other pharmacological inducers of ferroptosis may be efficacious, as it promotes cellular ferroptotic death through p62-NRF2 and autophagy flux inhibition in both migratory and bulk cancer cells [28].

The AKT/mammalian target of rapamycin (mTOR) signaling pathway is one of the most frequently mutated pathways in CRC that prevents cancer cells from oxidative stress and ferroptosis [28]. Activated AKT regulates cellular processes by phosphorylating a variety of intracellular proteins, such as the phosphorylation of NRF2 at tyrosine 568, which results in its translocation into the nucleus and the subsequent stimulation of the expression of anti-oxidant genes through p62-NRF2 antioxidant signaling [32,33]. Casein Kinase 1 (CK1) is a family of serine/threonine protein kinases that play a crucial role in various cellular processes, including cell proliferation, survival, and metabolism. Casein kinase 1 alpha (CK1α) is one of the most extensively studied isoforms of CK1 that simultaneously regulates Wnt/β-catenin and AKT/mTOR signaling pathways. Pharmacological inhibition of the CK1α as an inhibitor of AKT/mTOR signaling pathway has been investigated as potential therapy in preclinical studies. For example, selective inhibition of CK1α using D4476 inhibits autophagy flux, potentially suppressed the proliferation and the metastasis of RAS-mutated human CRC HCT116 cells, EGFR-mutant Non-small cell lung cancer (NSCLC), and human multiple myeloma U-266 cells. In addition, one study indicated that targeting CK1α can increase the sensitivity of colorectal cancer cells to 5-FU via the Wnt signaling pathway, G2 and S phase arrests, G1 arrest, reduction of ABCG2 mRNA, and inhibition of autophagy flux, potentially enhancing colon cancer treatment strategies [34]. It also determined that CK1α facilitates AML by inhibiting p53 downstream of MDM2-mediated autophagy and apoptosis, suggesting that targeting CK1α presents a therapeutic opportunity for AML treatment. Therefore, it concludes that selective inhibition of CK1α using D4476 can induce ferroptosis by the negative regulation of the p62-NRF2 axis alongside the inhibition of the autophagy. Consequently, we structure this study to assess whether Sal in combination with could influence the p62-NRF2 axis and ferroptosis through the CK1α signaling pathway in colorectal cancer cell lines [35].

## 2 Material and methods

### 2.1 Materials

The French company Biosera supplied RPMI-1640, fetal bovine serum (FBS), and penicillin-streptomycin. Sal was obtained from Sigma Aldrich (Germany), and D4476 (#120220) was purchased from Abcam (Cambridge, MA, USA). Sal and D4476 were administered at concentrations of 0.01–500 μM and 5 μM, respectively, after dissolution in DMSO.

### 2.2 Cell culture

The human HCT116 CRC cell line was acquired from the Iranian Pasteur Institute. The cells were incubated in RPMI-1640 medium containing 10 % bovine fetal serum, 100 units per milliliter of penicillin-streptomycin.

### 2.3 MTT assay

MTT (3-(4,5-Dimethylthiazol-2-yl)-2,5-Diphenyltetrazolium Bromide) assay was utilized to determine cell viability. The cells were cultured overnight in 96-well plates at a density of 4 × 103 cells/well. Then, they were exposed to Sal (0, 0.01, 0.1, 1, 10, 50, 100, and 500 μM) or D4476 (5 μM) for 24, 48, and 72 h. The crystals were then dissolved in 100 μL of DMSO. Using a microplate reader (Mikura Ltd.), the absorbance at 570 nm was determined.

### 2.4 RNA extraction and quantitative real-time PCR

Following the extraction of RNA from HCT116 cells (Kiyan Danesh Co., Shiraz, Iran), cDNA was synthesized using a cDNA Synthesis Kit (Cinaclon Co., Tehran, Iran). For the detection of mRNA levels, a SYBR green DNA PCR master mix (Ampliqon, Denmark) and an applied Biosystems 7500 real-time PCR system were employed. The comparative Ct method was used to assess mRNA expression, with GAPDH serving as the internal control. The sequences of the primers are provided in Table 1.

**Table 1:** The Primer sequences.

Genes	Forward primers (5′-3′)	Reverse primers (5′-3′)
***LC3 βII***	AACGGGCTGTGTGAGAAAAC	AGTGAGGACTTTGGGTGTGG
***P62***	AATCAGCTTCTGGTCCATCG	TTCTTTTCCCTCCGTGCTC
***Beclin-1***	AGCTGCCGGTTATACTGTTCTG	ACTGCCTCCTGTGTCTTCAATCTT
***MMP2***	TGA TGG CAT CGC TCA GAT CC	GGC CTC GTA TAC CGC ATC AA
***MMP9***	TCC CTG GAG ACC TGA GAA CC	CCA CCC GAG TGT AAC CAT AGC
***NRF2***	GCCATTAGTCAGTVGCTCT	GTGCCTTCAGTGTGCTTC
***PSAT1***	TGCCCAGAAGAATGTTGGCT	TCCAGAACCAAGCCCATGAC
***GAPDH***	CGACCACTTTGTCAAGCTCA	AGGGGTCTACATGGCAACTG

### 2.5 Scratch Wound Healing Assay

Typically, the scratch assay is used to quantify cellular migration on two-dimensional (2-D) surfaces in response to various treatments over time. Cells were seeded into 6-well plates (7 × 10^5^ cells/well) using a medium without serum. After 24 h, the cell monolayer was scratched with pipette tips. The wound was washed with PBS to eliminate non-adherent cells and treated with Sal or D4476 and their combination for 24 h. Migration of cells into the scratched region was detected using the microscope (Olympus Corporation, Tokyo, Japan) (40 × magnification) and analyzed using Image J software. The percentage of wound healing was calculated as:

% wound healing = (Area of wound (0 h) ‐ Area of wound (24 h))/Area of wound (0 h) × 100

### 2.6 Preparing cell lysates

The HCT116 cells were lysed during the final phase of the experiment. Using a trypsin/EDTA solution, the cells were extracted from the culture flask, neutralized with RPMI-1640, and centrifuged at 2000 RPM for 10 min. The resultant cell pellets were used to produce the lysates. For every 1 mL of PBS, 1.4 mg of protease inhibitor and 10 mL of phosphatase inhibitor were weighed out and added, respectively. Under the rule of 100 mL buffer (100 Triton X100 and 0.19 g in 100 cc water of EDTA 5 mM at pH = 7) for every 10^6^ cells, cell pellets were dissolved in lysis buffer. Cell lysates were flash-frozen in liquid nitrogen for 30 min, and then preserved at 80 °C before biochemical examinations were performed.

### 2.7 Biochemical measurement

The 10^6^ -cell lysates were thawed, centrifuged, and the supernatant was analyzed biochemically.

#### 2.7.1 Reduced glutathione (GSH) assay

The method for calculating the level of GSH conformed to Boulder's instructions [36]. Ellman's reagent disrupts the disulfide bond between thiols and forms 2-nitro-5-thiobenzoate (TNB), which ionizes in water at neutral and alkaline pH to form the TNB2 dianion.

The GSH concentration in the samples was determined by mixing 15 μL of hemolysates with 260 μL of assay buffer and 500 μL of Ellman reagents. After 15 min of incubation at the environment temperature, the generation of TNB2 was calculated using a spectrophotometer by measuring the absorbance of visible light at 412 nm. The absorbance quantities were matched to a standard curve derived from a well-established GSH standard curve.

#### 2.7.2 Lipid peroxidation assay

MDA, a marker for lipid peroxidation, was measured utilizing the Bioxytech MDA-586 assay test kit manufactured by the Zell Bio Company in Germany. At 45 °C, the absorbance of the reaction between MDA and N-methyl-2-phenylindole (NPMI) was determined to be 586 nm.

### 2.8 Statistical analysis

Multiple independent experiments are represented by their mean±standard deviations (SD). ANOVA and Tukey's post hoc test were used to evaluate the experimental results. The statistical calculations were performed with SPSS 24.00, and the significance level was set at *P* < 0. 05.

## 3 Results

### 3.1 How the combination of D4476 and sal helps to inhibit tumor growth?

**Fig. 1:**
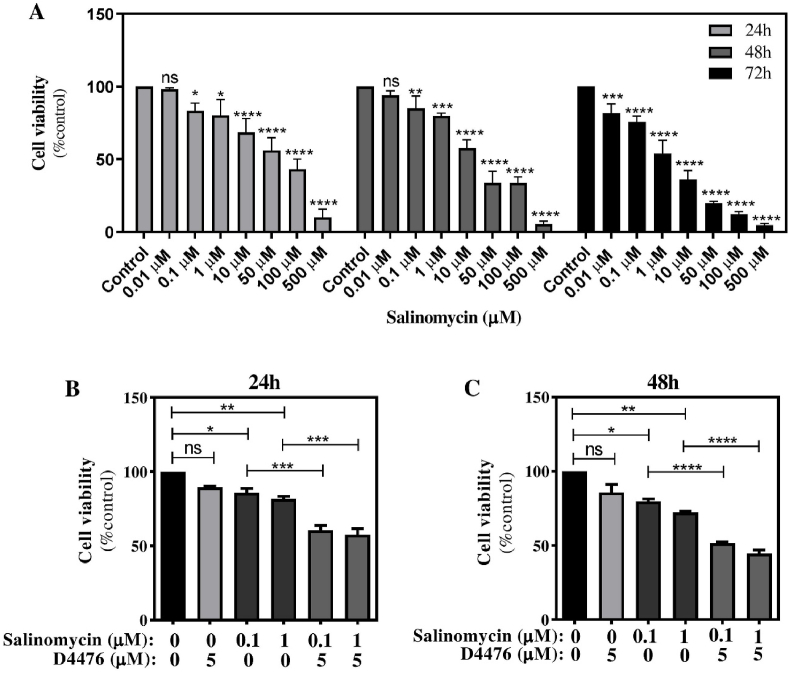
**The growth effects of Sal, D4476, and their respective combinations on HCT116 cells.** The cells were exposed to various Sal concentrations (A) and Sal in combination with 5 μM D4476 for 24 h (B) and 48 h (C). Data are reported as mean ± SD *(n = 3; ∗P < 0.05; ∗∗P < 0.01; ∗∗∗P < 0.001; ∗∗∗∗P < 0.0001).*

MTT assay was used to measure the human HCT116 CRC cell lineage growth after therapy with Sal alone or in combination with D4476. This was performed to determine if Sal and D4476 could prevent the growth of cancer cells. We found that Sal considerably decreased cell survival in HCT116 cells in 24, 48, and 72 h (Fig. 1A). Based on the MTT assay result, we selected Sal 0.1 and 1 μM for subsequent procedures at 24 and 48 h. Additionally, the combination of Sal (0.1 and 1 μM) and D4476 5 μM declined cell viability more effectively than Sal alone (p < 0.001) with the maximum reduction happening over 48 h (Fig. 1B–C) (*P* < 0.0001).

### 3.2 Sal treatment and CK1α inhibition alter autophagy pathway

**Fig. 2:**
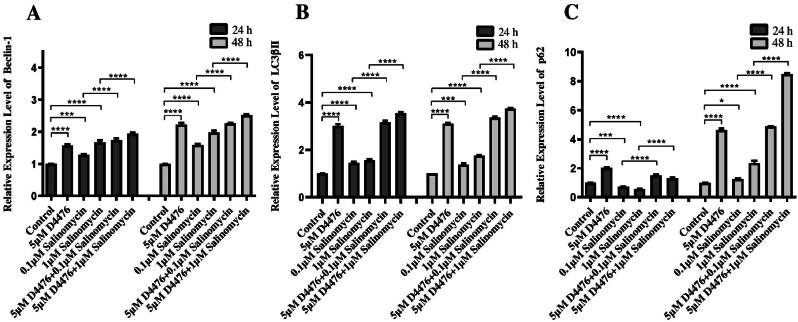
**The combination of Sal and D4476 led to intensified autophagy induction**. Levels of relative mRNA expression for *Beclin-1* (A), *LC3βII* (B), and *P62* (C) in HCT116 cells. *LC3βII*, *Beclin-1*, and *P62* relative mRNA expression levels were assessed. The results are presented as the mean ± SD of three separate experiments. *(∗P < 0.05; ∗∗P < 0.01; ∗∗∗P < 0.001; ∗∗∗∗P < 0.0001)*.

Subsequently, we employed real-time polymerase chain reaction to assess the impact of Sal alone and Sal in conjunction with D4476 on autophagy in HCT116 cells. HCT116 cells were exposed to either Sal alone (0.1 and 1 μM) or in combination with D4476 (5 μM) for 24 and 48 h. Based on Fig. 2A–C, D4476-treated groups exhibited significantly higher levels of *Beclin1* (1.57 and 1.58-fold at 24 and 48 h, respectively (, *LC3βII*, (3 and 3.1-fold at 24 and 48 h, respectively) and *P6*2 mRNA (2 and 4.63-fold at 24 and 48 h, respectively) compared to the control group (*P* < 0.0001). Moreover, both Sal alone and Sal in combination with D4476 were able to induce autophagy compared to control and Sal alone, respectively, by boosting the activity of the autophagy-related genes Beclin1 and LC3βII (*P* < 0.0001) after 24 and 48 h. As shown in Fig. 2C, a significant downregulation of P62 was observed in 0.1 (0.72-fold change) and 1 μM (0.56-fold change) Sal compared to the control (*P* < 0.001 and *P* < 0.0001, respectively) after 24 h, whereas the combination of Sal and D4476 could significantly up-regulate the expression level of P62 compared with 0.1 (2.08-fold change) and 1 μM (2.36-fold change) Sal alone (*P* < 0.0001).

### 3.3 Inhibition of CK1α suppresses migration in sal-treated HCT-116 cells

**Fig. 3:**
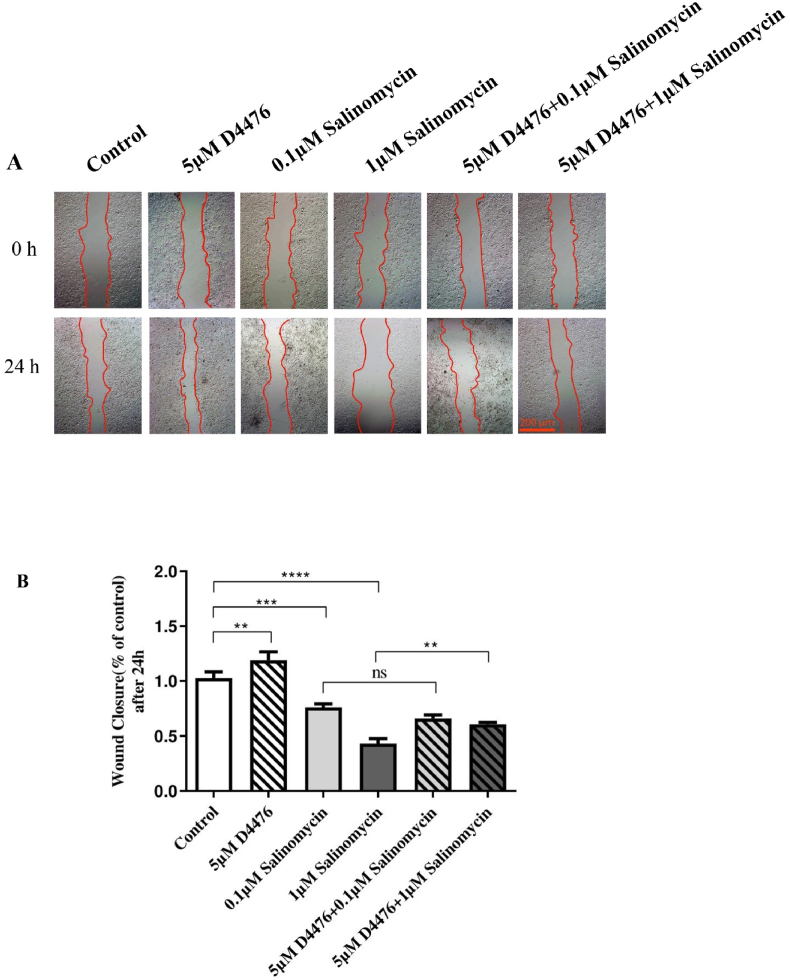
**Effect of Sal and its combination with D4476 on migration of the HCT116 colorectal cancer cell line (Scale bar:200 μm).** Monolayers of HCT116 cells were wounded and then treated with Sal and D4476. Using an inverted microscope, images were captured at 0 and 24 h, (A) (40 × magnification). For each concentration, scratch wound assays were carried out in triplicate wells. The results are presented as the mean ± SD of three separate experiments (B). *(∗P < 0.05; ∗∗P < 0.01; ∗∗∗P < 0.001; ∗∗∗∗P < 0.0001)*.

**Fig. 4:**
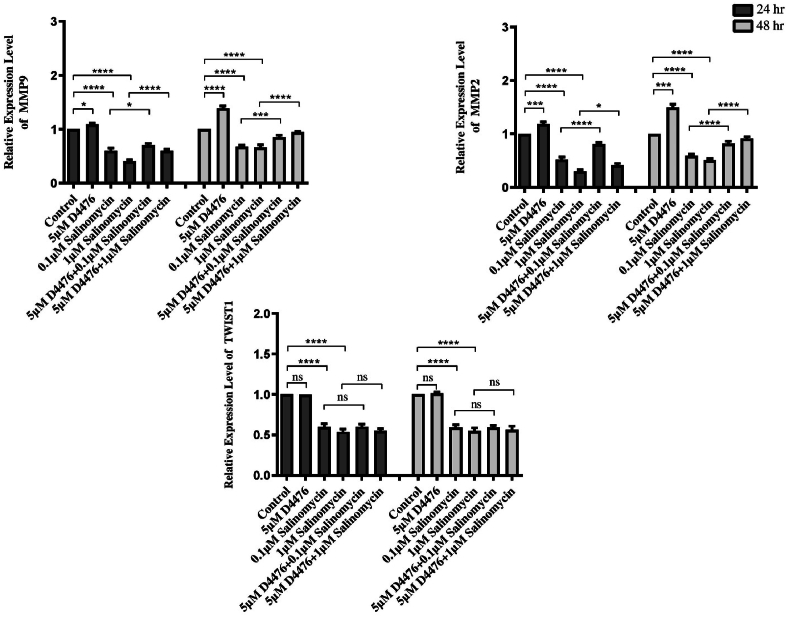
***MMP9*, *MMP2* and *TWIST1* relative mRNA expression levels in HCT116 cells.** The *MMP9*, *MMP2*, and *TWIST1* relative mRNA expression levels assessment was carried out in duplicate. The results are presented as the mean ± SD. *(∗P < 0.05; ∗∗p < 0.01; ∗∗∗P < 0.001; ∗∗∗∗P < 0.0001).*

In what manner could Sal regulate the migration of HCT116 cells independently or after D4476 treatment? Based on wound-healing assay, we find that Sal alone and in combination with D4476 stopped HCT116 cells from migrating much more than the control after 24 h (P < 0.0001, Fig. 3B). Moreover, the combination of 1 μM Sal with D4476 reduced HCT116 migration compared with 1 μM Sal alone (*P* < 0.01). Real-time PCR was used to examine the role of Sal and D4476 in HCT116 cell migration, with a focus on migratory-related genes such as Matrix metalloproteinase 2 (*MMP-2*), Matrix metalloproteinase-9 (*MMP-9*) and Twist-related protein 1 (*TWIST1*). *MMP-2* (1.18 and 1.49-fold at 24 and 48 h, respectively) *and MMP-9* (1.09 and 1.38-fold at 24 and 48 h, respectively) were upregulated following treatment with D4476 alone in comparison with control after 24 h and 48 h (Fig. 4).

Accordingly, 0.1 μM Sal alone can significantly decrease *MMP-2* (0.52 and 0.59-fold at 24 and 48 h, respectively)*, MMP-9,* (0.6 and 0.67-fold at 24 and 48 h, respectively) and *TWIST1* (0.59 and 0.58-fold at 24 and 48 h, respectively) mRNA expression compared with control (*P* < 0.0001). Moreover, 1 μM Sal alone can remarkably decrease *MMP-2* (0.3 and 0.51-fold at 24 and 48 h, respectively)*, MMP-9,* (0.4 and 0.66-fold at 24 and 48 h, respectively) and *TWIST1* (0.53 and 0.54-fold at 24 and 48 h, respectively) mRNA expression in comparison with control (*P* < 0.0001). Furthermore, co-treatment of Sal and D4476 noticeably enhanced the expression level of MMP-2 and MMP-9 mRNA compared with Sal alone. These findings suggest that Sal or its combination with D4476 can inhibit or even reverse the epithelial-mesenchymal transition (EMT) process in HCT116 cell lines.

### 3.4 What role does D4476 play in enhancing the effects of sal on HCT116 cell lines?

**Fig. 5:**
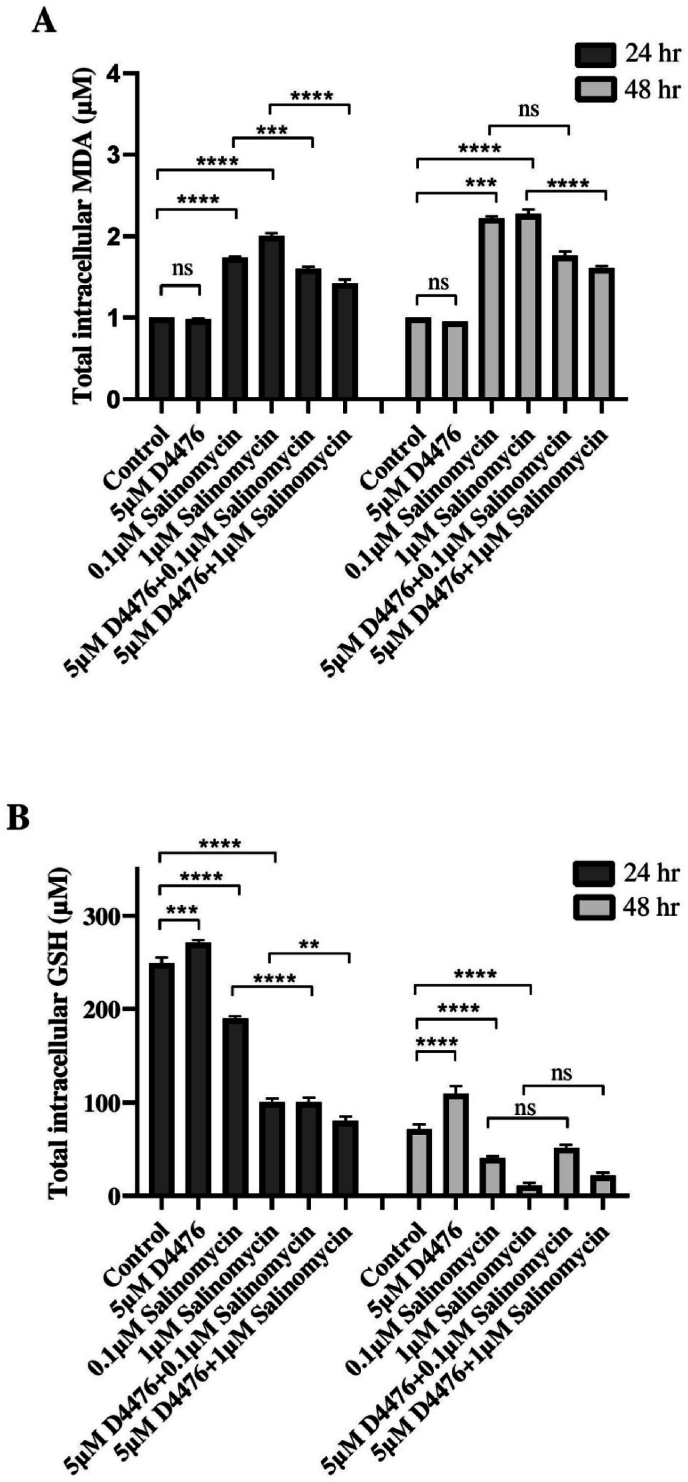
**MDA(A) and GSH(B) levels in HCT116 cells were measured using the respective *MDA* and *GSH* assays.** The data are expressed as the mean ± SD of three separate experiments. *,(∗P < 0.05; ∗∗P < 0.01; ∗∗∗P < 0.001; ∗∗∗∗P < 0.0001).*

Analysis of transcripts revealed that Sal alone or in combination with D4476 caused ferroptosis in RAS mutant HCT116 CRC cell lines. GSH and MDA levels, which are linked to ferroptosis, changed after treatment with Sal alone or in combination with D4476, depending on the dose and length of time. In comparison with control cells, MDA levels increased when cells were treated with 0.1 μM (1.96 and 1.19-fold at 24 and 48 h, respectively) and 1 μM (2.37 and 1.28-fold at 24 and 48 h, respectively) Sal alone (*P < 0.0001*, Fig. 5A), while GSH contents decreased following Sal treatment after 24 and 48 h (Fig. 5B). In terms of GSH levels, the D4476-treated groups demonstrated the opposite results relative to the control group. Compared to their corresponding Sal groups, all combination groups are less able to increase MDA levels (*P* < 0.0001), while GSH reduced in combination groups after 24h (*P* < 0.001).

### 3.5 Induction of ferroptotic cell death by sal and D4476

**Fig. 6:**
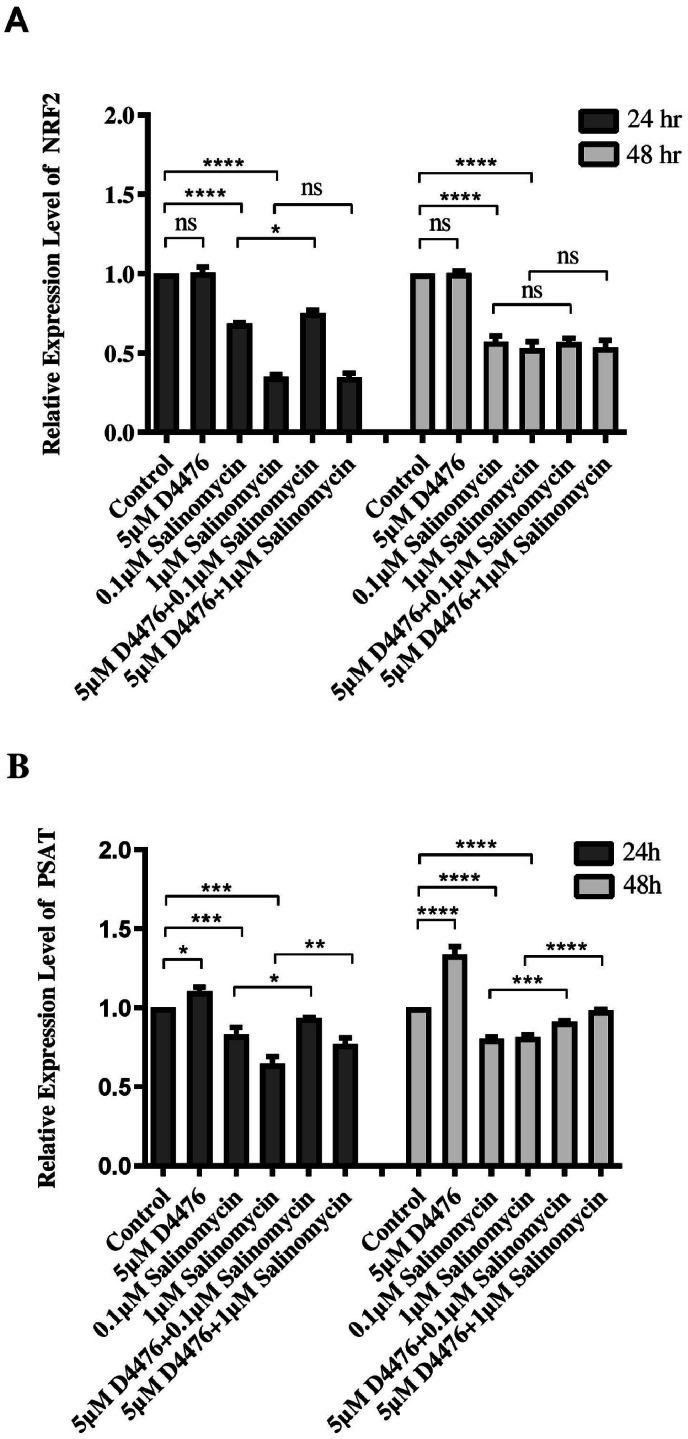
**The effects of Sal alone or in combination with D4476 on *NRF2* (A) and *PSAT1* levels (B).** The relative mRNA expression levels of *NRF2* and *PSAT1* were measured by Real-time PCR. The data is presented as the mean ± SD of three distinct experiments. *(∗P < 0.05; ∗∗P < 0.01; ∗∗∗P < 0.001; ∗∗∗∗P < 0.0001)*.

Real-time PCR was used to evaluate the effect of Sal and D4476 on the transcriptional activity and stability of *NRF2* and *PSAT1*. As shown in Fig. 6, a significant downregulation of *NRF2* and *PSAT1* was observed in Sal-treated cells compared to control after 24h and 48h (*P* < 0.001). The co-administration of Sal and D4476 significantly induced the expression level of PSAT1 mRNA in comparison to 0.1 μM (1.12 and 1.14-fold at 24 and 48 h, respectively) and 1 μM (1.19 and 1.21-fold at 24 and 48 h, respectively) Sal alone. NRF2 mRNA levels were significantly increased (1.1-fold change) after induction of cells with the combination of 0.1 μM Sal with D4476 compared to 0.1 μM Sal alone (P < 0.05). However, the use of D4476 alone did not result in a significant reduction in the transcriptional levels of NRF2 and PSAT. It appears that Sal alone is more effective than D4476.

## 4 Discussion

Our recent research showed that *CK1α* interferes with survival signaling cascades in CRC growth, and inactivation of *CK1α* synergistically with 5-fluorouracil induces cell death through autophagy flux inhibition [37]. We also found that Sal caused the HCT116 cell line to have more apoptosis by stopping autophagy flux and the UPR(29). Studies in vitro and in vivo have demonstrated that Sal inhibits colorectal cancer stem cells by inducing ferroptosis, although the mechanisms underlying its anticancer effects remain unclear [38,39]. Based on the discovery of Sal and *CK1α* ′s role in the regulation of the autophagic pathway in RAS-driven cancer, and in addition to the fact that autophagy and ferroptosis are two essential survival stress-management pathways for colorectal cancer, we evaluated the effect of Sal and *CK1α* inhibitor on autophagy and ferroptosis in HCT116 CRC cells in vitro by measuring cell survival, cell migration, and the expression levels of *MMP-2* and *MMP-9*, and autophagy-associated genes, *Beclin1*, *LC3βII*, and *p62*, along with the intracellular *MDA* concentration and *GSH/GSSG* ratio measurement.

Sal (0.1–1 μM) alone or in combination with D4476 (5 μM) decreased the viability of HCT116 cells in a concentration- and time-dependent manner. These results are consistent with prior research demonstrating that low Sal concentrations and high D4476 concentrations reduce the viability of tumor cells derived from osteosarcoma, pancreatic, leukemia, and breast cancer [37,40,41]. The in vitro anticancer properties of Sal and D4476 against HCT116 cell lines were determined by our research team previously [29,37]. To evaluate the combined anticancer effect of Sal and D4476, the viability of HCT116 cells was examined. HCT116 cell viability was significantly inhibited after two days of treatment with low concentrations of Sal and D4476. These in vitro outcomes are consistent with other studies showing that Sal or D4476 inhibits the growth of hepatocellular carcinoma, colorectal cancer, breast cancer, and pancreatic cancer in vivo [[42], [43], [44], [45], [46]].

*MMP* enzymes, such as *MMP-2* and *MMP-9,* promote extracellular matrix (ECM) degradation and basement membrane, thereby facilitating metastasis during the progression of cancer [46]. New treatment regimens are crucial for cancer treatment as metastases are the leading cause of death among cancer patients. In this situation, we illustrated that Sal or its combination with D4476 significantly decreased the expression of *MMP-2*, *MMP-9*, and *TWIST1*, thereby inhibiting in vitro cell migration in human CRC cells HCT116. These findings are in line with prior research explaining that Sal or D4476 inhibits breast cancer cell metastasis in vivo and prostate cancer cell migration in vitro in a selective manner, respectively [38,47,48].

Current research interests focus on the connection between autophagy and tumors. The *p62* protein is degraded during autophagy, and *p62*-deficient cells have higher basal levels of LC3*β*II, followed by an increase in autophagic function [49]. Treatment with Sal and inhibition of *CK1α* induced autophagy and increased *Beclin1*, *LC3βII*, and *p6*2 mRNA levels in the present study. *CK1α* inhibition with D4476 triggered the *β-catenin*/*TCF4's* (T cell-specific transcription factor) effect on *p62*, leading to *p62* suppression and autophagy induction [50,51]. The increased expression of cargo protein *p62* indicates that treatment with D4476 and Sal decreases autophagic flux, which accords with the findings of two earlier studies demonstrating the inhibition of the late stage of autophagy (autophagy flux) by D4476 and Sal in HCT116 cell lines [37,52].

Through the *p62 -NRF2* pathway, new research is showing that the accumulation of the autophagy marker p62 is a key part of what causes ferroptosis to happen. In conjunction with autophagy, the *Keap1-NRF2-ARE* pathway keeps cells safe from oxidative stress. In light of this, *Keap1-NRF2* may serve as a key link between ferroptosis and autophagy [26]. Tang et al. demonstrated that Sal inhibits the *p62- NRF2* pathway, consequently enhancing sorafenib-induced HCC cell ferroptosis [53]. Recent research has shown that initiation of the *NRF2/Keap1* signaling pathway protects head and neck cancer cell lines from cisplatin and artesunate-induced oxidative stress and apoptosis by positively controlling the levels of expression of the essential serine/glycine biosynthesis enzymes *PHGDH*, *PSAT1*, and *SHMT2*, whereas inhibition of this pathway reverses resistance to ferroptosis and enhances drug sensitivity [54]. It is interesting to note that among all amino acids, serine, as the primary precursor of glutathione, plays a vital function in colorectal cancer progression and chemoresistance, and that an increase in *PSAT1* enzyme expression after chemotherapy is associated with tumor progression in CRC patients [55]. Our investigation revealed that Sal alone or in combination with D4476 inhibited the *p62-keap1* interaction and suppressed the nuclear translocation of *NRF2*, resulting in decreased expression of downstream antioxidant *PSAT1* enzymes and induction of ferroptosis. Therefore, Sal, or D4476, is an *NRF2 inhibitor* and is involved in anti-colorectal cancer effects via the crosstalk between the *p62-NRF2* pathway and autophagy, which may provide novel insights into therapeutic directions for colorectal cancer based on the anti-oxidant response during tumor progression.

Glutathione is a required cofactor for Glutathione peroxidase 4 (*GPX4*) and plays a crucial role in the process of ferroptosis. Recent research reveals that *GSH* depletion additionally influences autophagy, as shown by an increase in the expression of *LC3βII*, autophagic vacuoles, and autophagic flux [56]. Selective inhibitors of ferroptosis (such as *Fer-1* and *Lip-1*) [45] and autophagy inhibitors like Sal or D4476 in the present study prevented cellular death dependent on GSH depletion that was triggered by promoting *GSH* depletion and *MDA* production. Autophagy decreases intracellular *GSH* levels significantly but increases the levels of lipid peroxidation products such as *MDA*, *ROS*, and 4-Hydroxynonenal (*4-HNE*) [57]. Presumably, the mutual effects of *GSH* and *MDA* in conjunction with autophagy modulate ferroptosis induction.

## 5 Conclusion

This study demonstrated the novel antitumor activity of Sal in combination with D4476 in HCT116 CRC cell lines by interfering with the accumulation of lipid peroxidation products and triggering an imbalance in redox homeostasis. Moreover, our research revealed the inhibitory effect of Sal or its combination with D4476 on the anti-oxidant *p62-NRF2-PSAT1* pathway following autophagic flux inhibition required for ferroptosis induction. Future research will concentrate on the in vivo effects of Sal in conjunction with the D4476 or *p62-NRF2-PSAT1* pathway in CRC tissues. Our discovery would offer a novel strategy for treating CRC.

## Ethical approval

The present study was designed and conducted with the permission of the Ethics Committee of Shiraz University of Medical Sciences (IR.SUMS.REC.1401.453).

## Consent to participate and consent to publish

Not applicable.

## CRediT authorship contribution statement

**Sara Khakshournia:** Writing – original draft. **Morvarid Siri:** Writing – original draft, Writing – review & editing. **Mozhdeh Zamani:** Visualization. **Farzaneh Bozorg-Ghalati:** Data curation, Formal analysis. **Zahra Mojtahedi:** Conceptualization, Software. **Somayeh Igder:** Methodology. **Negar Azarpira:** Investigation. **Pooneh Mokarram:** Funding acquisition, Project administration, Supervision, Validation, Visualization.

## Declaration of competing interest

The authors have no conflict of interest to declare.

## Data Availability

Data will be made available on request.
